# Egg Eviction Imposes a Recoverable Cost of Virulence in Chicks of a Brood Parasite

**DOI:** 10.1371/journal.pone.0007725

**Published:** 2009-11-11

**Authors:** Michael G. Anderson, Csaba Moskát, Miklós Bán, Tomáš Grim, Phillip Cassey, Mark E. Hauber

**Affiliations:** 1 Ecology and Conservation Group, Institute of Natural Science, Massey University, Albany Campus, Auckland, New Zealand; 2 Animal Ecology Research Group of the Hungarian Academy of Sciences, Hungarian Natural History Museum, Budapest, Hungary; 3 Behavioural Ecology Research Group, Department of Evolutionary Zoology, University of Debrecen, Debrecen, Hungary; 4 Department of Zoology and Laboratory of Ornithology, Palacky University, Olomouc, Czech Republic; 5 Centre for Ornithology, School of Biosciences, University of Birmingham, Edgbaston, United Kingdom; 6 Department of Psychology, Hunter College, City University of New York, New York, United States of America; University of Lethbridge, Canada

## Abstract

**Background:**

Chicks of virulent brood parasitic birds eliminate their nestmates and avoid costly competition for foster parental care. Yet, efforts to evict nest contents by the blind and naked common cuckoo *Cuculus canorus* hatchling are counterintuitive as both adult parasites and large older cuckoo chicks appear to be better suited to tossing the eggs and young of the foster parents.

**Methodology/Principal Findings:**

Here we show experimentally that egg tossing imposed a recoverable growth cost of mass gain in common cuckoo chicks during the nestling period in nests of great reed warbler *Acrocephalus arundinaceus* hosts. Growth rates of skeletal traits and morphological variables involved in the solicitation of foster parental care remained similar between evictor and non-evictor chicks throughout development. We also detected no increase in predation rates for evicting nests, suggesting that egg tossing behavior by common cuckoo hatchlings does not increase the conspicuousness of nests.

**Conclusion:**

The temporary growth cost of egg eviction by common cuckoo hatchlings is the result of constraints imposed by rejecter host adults and competitive nestmates on the timing and mechanism of parasite virulence.

## Introduction

**Figure 1:**
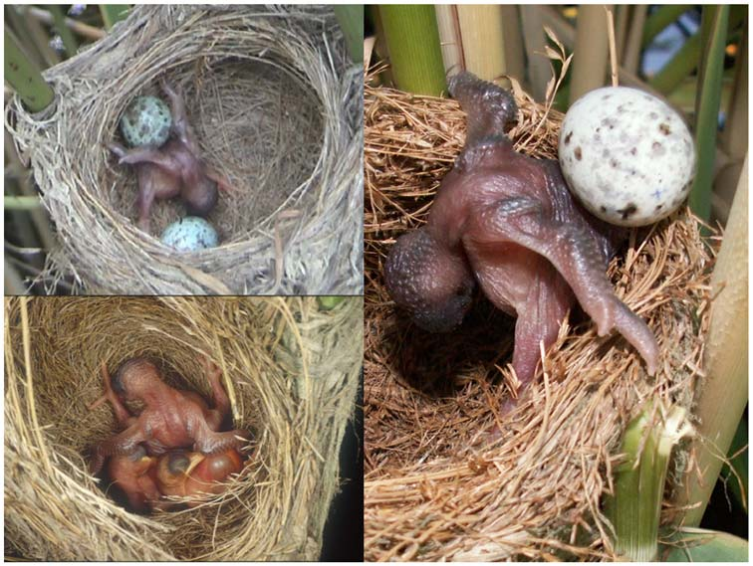
Hatchling common cuckoos in the process of evicting host eggs and chicks from great reed warbler nests. Photo credits from M. Honza (upper left), M. Bán (right), and C. Moskát (lower left).

The remarkable ability of the common cuckoo hatchlings *Cuculus canorus* (hereafter: cuckoo) to evict host eggs and nestmates from the nest (Fig. 1) has fascinated naturalists since the time of Aristotle [1], [2] but was first documented in the scientific literature much later – about 220 years ago [3]. Eviction represents a virulent behavioral strategy to eliminate costly competition with nestmates [4], [5], [6]. Yet both the mother parasites, that remove one or more host eggs when laying her own egg [7], and older cuckoo nestlings, that are larger and beg more intensely than host chicks [8], appear to be better equipped to eliminate eggs or cohabiting nestmates. Why does it then fall to the naked and blind cuckoo chick to complete the task of tossing eggs and hatchlings over the rim of the host nest?

In general, how eviction behavior in brood parasite nestlings evolved is poorly understood. One suggestion postulated by Soler [9], [10] is that parasite virulence is determined by the breeding strategy of the host species. Two main breeding strategies have been described for parent birds: 1) clutch size adjustment and 2) brood reduction. Clutch size adjusters allocate food evenly amongst nestlings, and even preferentially feed young that are in poorer condition, so that all members of the clutch fledge. Alternatively, in brood reducers, parents lay larger clutches than they are capable of raising, reducing the brood at the later stages by selectively feeding larger nestlings. Soler [10] suggested that this could act as a mechanism to drive the evolution of eviction behavior, as sole brood parasite nestlings in nests of brood reducer species can survive better. By contrast, cuckoo nestlings in nests of clutch size adjuster hosts will not receive increased parental provisioning with increased begging intensity, and might even be less likely to survive to fledge. Therefore, it is likely that the evolution of eviction behavior was necessary for cuckoos parasitizing clutch adjuster species. To evaluate these scenarios requires answering the many questions regarding the dynamics and the costs of eviction behavior that need to be overcome before such a behavior could evolve. Aspects of the fitness-relevant dynamics of eviction behavior include reduced growth due to energetic costs and reduced time spent begging, as well as the potential for increased predation rates [11], [12].

Previous work revealed that the timing of virulence is prohibitively constrained by hosts because single egg clutches of foreign (parasitic) eggs are typically abandoned and rejected by foster parents [13], [14]. Similarly, if cuckoo chicks were to cohabitate with host nestmates, they would face permanently costly competition for foster parental care [15] and suffer from lower growth [5], [6], [15] or very high mortality [5], [16]. Therefore, the window of virulence by cuckoo parasites appears to be open only briefly after the cuckoo chick hatches [12].

The benefits of eviction are clear in that cuckoo chicks receive parental care without competition and grow and survive better [6], [12]. However, the costs of egg eviction relative to egg removal by mother parasites and competition with host nestmates within the same species remain undescribed to date. In a separate set of experiments, which included returning evicted artificial eggs throughout the egg evictor phase of cuckoo chicks' development, we have recently demonstrated temporary growth costs and delayed fledging owing to evicting eggs in nests of a common host of the cuckoo, the redstart *Phoenicurus phoenicurus*. Correlational data from the same study suggested that nest architecture also influences the cost of eviction [12], [17]. Nevertheless, in this context the redstart may be atypical because it is the only common cavity breeding cuckoo host, and parasite chicks often fail to successfully eliminate nestmates and die as a consequence.

Here, we examined the generality of the hypothesis that eviction behavior incurs a moderate and recoverable cost in a typical open-nesting host of the cuckoo. We studied cuckoos that hatched in the deep nests of a relatively large host [18], [19], the great reed warbler *Acrocephalus arundinaceus*, and measured differences in growth rates between hatchlings that evicted natural nest contents and those whose nests were experimentally emptied. We tested two specific hypotheses; 1) the “ghost of eviction past” and 2) “compensatory growth” hypothesis. The “ghost of eviction past” hypothesis predicts poorer growth performance of evictor chicks compared to non-evictor chicks, continuing after the eviction instinct ceases. It may also lead to a possible growth pattern, in which growth rate is equivalent, but ontogenetically delayed, which would lead to the same fledging mass, but an older fledging age [12]. Alternatively, the “compensatory growth” hypothesis predicts that evictor chicks, even if experiencing early growth costs of eviction, are able to recover their growth in the latter parts of the nestling period to fledge at similar masses as non-evictor chicks. We predict that eviction will differentially affect growth of mass (decrease) and structures involved in begging (no effect or increase, see [20]) Finally, we also compared predation rates between non-evictor and evictor nests to test the prediction of the hypothesis that evictor behavior is costly because it is more conspicuous as tossed eggs attract more predators.

## Methods

### Field Procedures

Research was conducted in Hungary, about 30–40 km south of Budapest, in the regions of Apaj and Kiskunlacháza (47°09′, 19°05′). Great reed warblers breed at these sites in reed *Phragmites australis* beds that grow in 2–4 m wide margins of small channels and experience an unusually high level of parasitism (41–68% nests per year: [21]). Field work was conducted from mid-May to mid-July 2008. Host nests were monitored daily during the laying period and again at around the expected hatching dates. Parasitized nests with a single cuckoo egg were randomly assigned at hatching into one of two treatments. In *evictor* nests, we left the host clutch in the nest and allowed cuckoo nestlings to evict host eggs naturally. In *non-evictor* nests we removed all host eggs to eliminate eviction behavior. Our research followed guidelines of the Animal Behavior Society for the ethical use of animals in research and permission for the fieldwork was provided by the Hungarian Inspectorate for Environment, Nature and Water Resources.

To analyze differences in the development of cuckoo nestlings, we quantified growth rates using several parameters (mass, tarsus, gape length, gape width). Importantly, although these measures are generally intercorrelated they cannot be combined into a single measure of growth because they may be subject to a variety of life history trade-offs [22]. For instance, Gil et al. [20] showed that chicks in poorer condition might invest more into structures that serve to increase provisioning (e.g. gape area). Accordingly, we calculated gape area because it is one of the factors known to be involved in soliciting sufficient parental resources for the fast growing cuckoo chick [23].

Nestling mass was measured using portable electronic scales (precision: 0.01 g) and morphological measurements were taken using Vernier calipers (precision: 0.05 mm). We measured gape length (GL) from the outside edge of the rictal flange to the tip of the bill and gape width (GW) was the maximum distance between the outer corners of the rictal flange. These two measurements were used to estimate of gape area (GA). We calculated gape area using the formula: 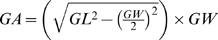, assuming that the maxilla and mandible of cuckoo nestlings are of equal area and that the shape of each is triangular (see [23]).

### Sample Sizes

Nests were assigned to evictor (n = 21) and non-evictor (n = 17) treatments and checked subsequently in a random order. We confirmed that all host eggs were evicted from all evictor nests. Clutch sizes (host and parasite eggs combined) were similar between treatment groups (mode: 5 eggs, range 3–6, t-test, *t*_30_ = 1.30, p = 0.20). We attempted to take measurements every day, but were occasionally unable to do so due to inclement weather; thus, the numbers of measurements per nestling are variable. Overall, the dates when measurements were taken for the two treatment groups were also similar: median for evictor  = 13^th^ June (n = 228), non-evictor  = 15^th^ June (n = 149; generalized linear mixed model, controlling for chick identity: *F*_1,38.1_ = 0.44, *p* = 0.51). Also, the number of nestlings decreased with age due to predation.

### Data Analyses

Comparing growth data presents statistical problems for standard linear model techniques because the sigmoid growth patterns of birds violate the assumption of linearity of effects and homogeneity of variance [24]. Therefore, we analyzed the deviations of growth parameters from evictor cuckoo chicks (i.e., developing under natural conditions), rather than raw growth data. The aim of this approach was to obtain estimates of chick growth performance that would not violate the assumption of linearity of generalized linear mixed models (GLMM). We thus compare data between two treatment groups against a common growth curve model (see below), and so the type of growth curve selected would not affect the direction of differences between residuals.

In our analyses, for mass data we first fitted logistic growth curves (PROC NLIN in SAS with the Levenberg-Marquardt estimation method; see [24]) to data from evictor chicks; to reduce pseudoreplication one random measurement per chick was used to generate this growth curve. The resulting logistic curve had following parameters: mass(t) = 87.66/(1+e^(−0.35*(t–8.20))^) (t  =  chick age in days). We then calculated differences between observed chick masses and those predicted by this standard growth curve (i.e., residuals). Thus, positive residual values designate better growth performance of an individual chick compared to the average evictor chick. Data for structural growth were best fitted by second order polynomial regressions in all cases as follows:

Tarsus (t)  =  11.61 + 0.82*t – 0.04*t^2^

Gape length (t)  =  10.87 + 0.96*t – 0.03*t^2^

Gape width (t)  =  11.82 + 0.46*t – 0.04*t^2^

Gape area (t)  =  99.42 + 20.80*t – 0.70*t^2^

The calculated growth parameters, i.e. residuals, were then analyzed using GLMM (PROC MIXED module in SAS; normal error distribution, parameters estimated by REML, denominator degrees of freedom were calculated using the Kenward–Roger method). We used the variance components covariance structure in all models. Models had nest ( = cuckoo chick) identity as a random factor, treatment (evictor vs. non-evictor) as nominal predictor and chick age as continuous covariate. Age was a significant factor in some nestling periods (see below) and so we conservatively controlled for it in all models. However, the removal of age did not affect results qualitatively in any model; treatment*age interactions were always non-significant (all P>0.05) and removed in all cases. All models were checked for the linearity of effects, normality of errors and homogeneity of variances and were found satisfactory [25].

Honza et al. [11] showed that cuckoo chicks in great reed warbler nests start to evict hosts eggs on average 2 days after hatching. Therefore, we began our analyses of the differences between non-evictor and evictor nestlings during this initial period. Eviction instinct typically disappears when cuckoo chicks are 5 days old [1], [6] although can last until later in other species [12]. Therefore, we analyzed growth data during the periods from 3 to 5 and 6 to 8 days of age posthatch. Based on these periods, we divided the totality of the nestling period into 3-day phases, prior and subsequent to eviction, for further statistical comparisons between treatment groups. We estimated chick fledging age as a mid-point between the last nest check when the chick was in the nest and the first nest check when the nest was empty and there were no signs of predation.

Although we made repeated comparisons between evictors and non-evictors across different periods (Table 1), a Bonferroni correction is generally considered unsuitable for ecological studies as it increases a risk of type II error ([26] and references therein). Further, we did not test for *any and all* differences between age groups but our predictions were both temporally and directionally specific. Under such conditions the use of Bonferroni corrections would be not applicable.

**Table 1:** Differences in growth parameters between non-evictor (chicks raised alone, host eggs removed) and evictor (host eggs left and evicted) cuckoo chicks in great reed warbler nests.

Variable	Phase	Effect size	Sample size		F	df	P
	(days)		chicks	measurements			
*Mass*	0–2	0.07±0.37	31	68	0.03	29.5	0.86
*(g)*	**3–5**	**2.42±1.04**	**32**	**75**	**5.47**	**30.2**	**0.026**
	**6–8**	**4.76±1.99**	**22**	**60**	**5.73**	**19.9**	**0.027**
	9–11	1.49±2.40	22	60	0.38	19.4	0.54
	12–14	3.22±2.95	21	53	1.20	18.7	0.29
	15+	3.04±2.28	21	52	1.77	17.4	0.20
*Tarsus*	0–2	0.16±0.22	32	46	0.55	19.1	0.47
*(mm)*	3–5	0.41±0.31	32	64	1.73	25.7	0.20
	6–8	0.55±0.38	23	55	2.06	19.0	0.17
	9–11	0.13±0.52	22	57	0.06	19	0.80
	12–14	0.25±0.37	21	49	0.45	18.1	0.51
	15+	0.19±0.44	17	50	0.19	13.9	0.67
*Gape*	0–2	−0.58±0.36	32	47	2.67	25	0.11
*length*	3–5	−0.02±0.37	32	67	0.00	27.8	0.97
*(mm)*	6–8	0.32±0.46	23	55	0.49	18.9	0.49
	9–11	0.10±0.43	22	59	0.05	19.6	0.83
	12–14	0.30±0.40	21	49	0.56	17.5	0.46
	15+	−0.02±0.42	17	49	0.00	14	0.97
*Gape*	0–2	−0.03±0.25	32	46	0.01	22.2	0.92
*width*	3–5	0.19±0.25	32	67	0.55	26.9	0.47
*(mm)*	**6–8**	**0.75±0.33**	**23**	**55**	**5.13**	**19.1**	**0.035**
	9–11	0.32±0.31	22	59	1.05	20.2	0.32
	12–14	0.46±0.34	21	50	1.79	17.1	0.20
	**15+**	**0.55±0.24**	**17**	**49**	**4.98**	**11.1**	**0.047**
*Gape*	0–2	−6.05±5.26	32	46	1.32	22.8	0.26
*area*	3–5	2.34±7.03	32	67	0.11	28	0.74
*(mm^2^)*	6–8	14.74±10.82	23	55	1.86	19	0.19
	9–11	6.28±10.42	22	59	0.36	19.6	0.55
	12–14	12.65±12.03	21	49	1.11	17.4	0.31
	15+	10.76±9.83	17	49	1.20	14.1	0.29

Data from *a priori* defined phases of development were analyzed separately. Growth was estimated as deviations from growth patterns of evictor chicks randomly sampled in the study population (see Methods). Effect size (mean ± SE) is the difference between the growth parameter of non-evictor and evictor groups (i.e., positive effect  =  greater growth of non-evictor chicks). Sample sizes for respective periods are given as number of nests/chicks and measurements and df refers to denominator degrees of freedom from GLMM models controlling for chick identity and age.

We did not manipulate number of eggs in the nests with evictor cuckoo chicks. Thus, the number of evicted eggs naturally varied from 2 to 5. We therefore tested the correlation between the number of eggs ejected on the growth rates of nestlings within the evictor group. The same structure of GLMM that tested for the effect of eviction versus non-eviction on growth was used, but with the number of eggs evicted as the fixed effect, while maintaining nest (cuckoo chick) as a random variable and age as a covariate. We set α = 0.05 and report effect sizes for both significant and non-significant comparisons [27].

## Results

**Figure 2:**
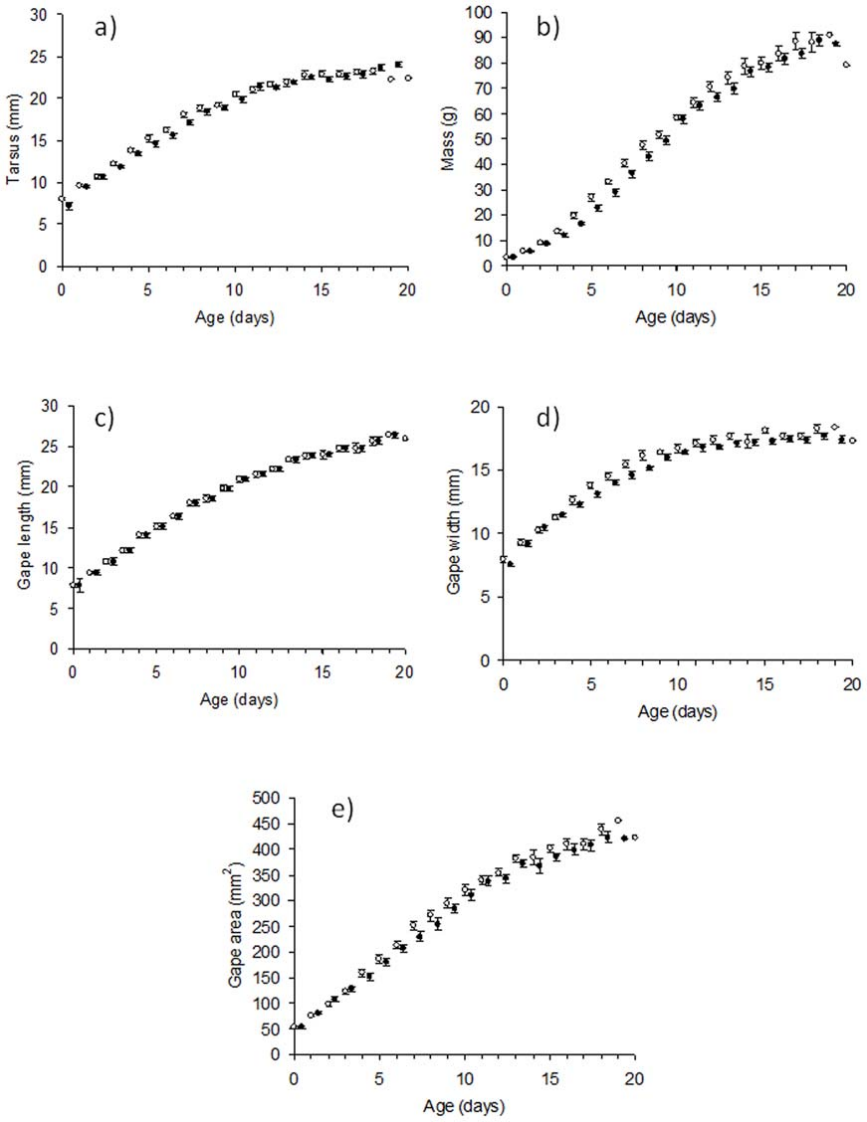
Growth of common cuckoo chicks in great reed warbler nests with host eggs left that had to be evicted by cuckoo chicks (black circles: evictor group) or where host eggs were removed (open circles: non-evictor treatment). For a) mass, b) tarsus, c) gape length, d) gape width, e) gape area. Values are means ± SE.

Growth parameters of cuckoo hatchlings in the non-evictor treatment were statistically identical to those of the evictors during the period prior to the onset of eviction (non-evictor/evictor ratio: 92–103%, referring to the growth of non-evictor chicks in relation to evictor, i.e. 100% is equal growth and more than 100% is a faster growth rate for evictor chicks) (Table 1, Fig. 2). However, during and immediately following the eviction phase (days 3–5 and 6–8), non-evictor cuckoo chicks grew at a faster rate than evictors with respect to mass (110–120%: Table 1 and Fig. 2a). From day 9 until fledging, the differences between the two treatment groups were non-significant in all comparisons (Table 1).

As predicted by the compensatory hypothesis, the mass gain of non-evictor chicks became similar to evictors prior to fledging. This result was obtained by comparing the last measured weight of chicks prior to fledging (evictors: 84.8±1.88 g, non-evictors: 85.6±2.76 g, U_7,7_ = 0.13, *p* = 0.90). Evictor and non-evictor chicks were last weighed at similar ages prior to fledging (days 17–20; evictor: 18.0±0.43 vs. non-evictor: 18.3±0.36, U_7, 7_ = 0.61, *p* = 0.54). There was no statistical difference in fledging ages between the two groups (evictor: 18.11±0.44 days vs. non-evictor: 19.0±0.48 days, U_9, 6_ = 15.5, *p* = 0.17).

Although in most comparisons tarsus, gape length, gape width, and gape area were greater for non-evictor than evictor chicks (Fig. 2b–e, Table 1), in contrast to mass data, these morphological measurements were highly variable between treatment groups, so that only two of the differences reached statistical significance (Table 1).

The rate of mass gain of cuckoo nestlings during the nestling period differed amongst those that evicted differing number of eggs (Table 2). Our correlational data showed that the mass (g) of nestlings that evicted 5 eggs was significantly greater than those that only evicted 2, 3, or 4 eggs (2 vs 5, mean difference ± s.e.: −9.38±4.16, df = 13.08, *p* = 0.042; 3 vs 5, −8.301±2.98, df = 17.2, *p* = 0.013; 4 vs 5, −7.54±2.42, df = 14.82, *p* = 0.007). There was no significant difference amongst nestlings that evicted 2, 3 or 4 eggs (all *p*>0.05). No other measures of growth correlated amongst evictor nestlings with the number of eggs evicted (Table 2).

**Table 2:** The effect of the number of eggs evicted by cuckoo nestlings (n = 20) on growth parameters within the evictor group for the nestling period.

Variable	Effect Size	Measurements	F	df	P
**Mass (g)**	**6.08**±**2.24**	**206**	**3.80**	**15.19**	**0.03**
Tarsus (mm)	11.33±0.46	173	3.38	9.98	0.06
Gape Width (mm)	10.44±0.4	180	1.85	12.48	0.19
Gape Length (mm)	10.96±0.6	180	1.27	15.25	0.32
Bill Area (mm^2^)	84.71±12.35	179	1.23	16.07	0.32

Growth was estimated as deviations from growth patterns of evictor chicks randomly sampled in the study population (see Methods). Effect sizes (mean ± SE) refer to the regression coefficients for each model. Sample sizes for respective periods are given as number of measurements, and df refers to denominator degrees of freedom from the GLMM model controlling for chick identity and age.

The predation rates of non-evictor vs. evictor groups (3 of 14 nests and 8 of 15 nests, respectively) were not significantly different (Fisher's exact test, *p* = 0.13).

## Discussion

Parasitic chicks of the typically evictor common cuckoo experience a temporary reduction of mass gain following the elimination of host progeny in nests of the great reed warbler. Still, we detected no permanent costs during the nestling period in this experiment on the natural range of virulence by the hatchling parasite, including potential delayed fledging [12] or predation costs [28]. At the same time we did not test for costs that may impact birds during later stages of their life-history [29], as compensatory growth patterns are known to cause stress during nestling development which may lead to oxidative damage [29], reduced immunocompetence [30], and even loss of cognitive abilities in adulthood [31] (see [32] and [33] for reviews). These types of costs may have been missed by us as it would have required data across longer periods, including overwinter survival [34], [35]. Also, future work could use comparative studies between host populations [36] and across different host species [37] or experimental manipulations to gage the generality of the (lack of) realized costs of eviction by manipulating the size, weight, or number of the evicted eggs in parasitized nests, changing the nest architecture to change the cost of eviction, or exposing evictor cuckoo chicks to host hatchlings rather than eggs [12].

Using the current data, our results appear to conform to the compensatory growth hypothesis, as there were no differences between evictor and non-evictor nestlings during the late stages of the nestling period, suggesting that cuckoo chicks are able to increase their rate of mass gain following the eviction period. None of the other morphological variables measured indicated a consistent reduction in growth due to the eviction process. Of particular interest is that bill dimensions were similar between evictor and non-evictor cuckoo chicks. Thus, reduced mass gain was not paralleled by a reduced development rate of the gape area, suggesting that increased allocation may have been channeled towards gape growth relative to mass [20], so as to maintain an adequate visual signal of need [23]. Compensatory growth [38] may occur if foster parents are able to compensate the growth reduction of evictor cuckoo chicks. This is suggested by our counterintuitive correlational data on cuckoo chick growth. Specifically, we found that cuckoo chicks evicting 5 host eggs grew faster than cuckoo chicks evicting fewer eggs (Table 2). Such a result is consistent with a pattern of better parental care by foster parents who are also able to lay larger clutches (also see [39], [40], [41]). Alternatively, female cuckoos may be preferentially laying and removing fewer host eggs from nests with other indicators of higher parental ability, including nest defense or nest size [39].

Kilner [42] applied the use of a cost/benefit model to explain variation in nestling virulence. Under this model, whenever the costs of sharing a nest with nestmates are greater than any potential benefits, such as an increase in the production of begging signals owing to larger number of nestmates [43], then eviction behavior should evolve. Our study supports the assumption that the costs of eviction behaviors are biologically realized. In turn, even temporary costs of virulence might alter the threshold where it becomes beneficial for the parasite chick to be raised alone [44], resulting in host-parasite systems, where alternative strategies of virulence will be employed, such as increased competitiveness with host nestlings or direct killing of nestmates by hatchling parasites [1].

We suggest that timing of eviction by the naked and blind cuckoo chick can be explained by an ongoing coevolutionary arms race between hosts and parasites [45], whereby hosts escalate to evolve increasingly specialized responses to reduce the cost of parasite adaptations to circumvent rejection [46]. Overall, (1) the potential strategy of the early removal of future competitors at the *egg* stage by female cuckoos leads to unrecoverable costs (e.g., the desertion of parasitized nests by hosts: [14]), (2) the potential strategy of late removal of competitors at the *chick* stage by the typically older and larger cuckoo chick also leads to unrecoverable costs (e.g., impaired growth, survival and fledging of the parasite chick caused by costly competition with host chicks: [5], [6], so that (3) eviction by the blind and naked cuckoo chick remains the only feasible option for the cuckoo to become the sole occupant of the host nest [3]. Nevertheless, this cost of early eviction is temporary, recoverable, and compensated for later in the nestling period in broods of great reed warbler hosts (this study, 35). The cost of eviction is also likely to vary with the size of host eggs and nestlings, as well as the nest structure [12], [17]. Our study may not be indicative of all of the costs of eviction, as 1) great reed warbler eggs are larger than typical host eggs, increasing the cost of eviction and 2) the eviction of eggs is likely to be easier than that of host nestmates, thus underestimating biologically realized eviction costs. Finally, the mechanisms of compensatory growth, including possible increases in the cuckoo chicks' signaling of need for parental provisioning following egg tossing, still remain to be elucidated.
